# On a Blockchain-Based Security Scheme for Defense against Malicious Nodes in Vehicular Ad-Hoc Networks

**DOI:** 10.3390/s22145361

**Published:** 2022-07-18

**Authors:** Guandong Liu, Na Fan, Chase Q. Wu, Xiaomin Zou

**Affiliations:** 1 School of Economics and Management, Chang’an University, Xi’an 710064, China; liuguandong@chd.edu.cn; 2 School of Information Engineering, Chang’an University, Xi’an 710064, China; fnsea@chd.edu.cn (N.F.); 2020124138@chd.edu.cn (X.Z.); 3 Department of Data Science, New Jersey Institute of Technology, Newark, NJ 07102, USA

**Keywords:** vehicular networks, detection of messages, blockchain, consensus mechanism, privacy protection

## Abstract

Vehicular ad-hoc networks (VANETs) aim to provide a comfortable driving experience. Sharing messages in VANETs can help with traffic management, congestion mitigation, and driving safety. However, forged or false messages may undermine the efficiency of VANETs. In this paper, we propose a security scheme based on blockchain technology, where two types of blockchain are constructed based on roadside units (RSUs) and Certificate Authorities (CAs), respectively. The proposed security scheme has multifold goals to identify malicious nodes and detect forged messages based on multiple factors, such as reputation of sender nodes, and time and distance effectiveness of messages. In addition, an incentive mechanism is introduced on the RSU blockchain to encourage RSUs to adopt active behaviors. Extensive simulations show that the proposed scheme exhibits superior performances to existing methods in detecting forged messages and identifying malicious nodes. Meanwhile, it provides privacy protection and improves the efficiency of vehicular networks.

## 1. Introduction

It is estimated that the total number of registered vehicles will reach two billion within the next 10 to 20 years [1]. Vehicular ad-hoc networks (VANETs) has been considered as the foundation of an intelligent transportation system (ITS) to improve transportation efficiency and ensure the safety of both vehicles and drivers. There are two types of communications in VANETs, namely, vehicle-to-vehicle (V2V) communication and vehicle-to-infrastructure (V2I) communication, which are carried out to facilitate cooperation and sharing among vehicles and RSUs.

Compared with the traditional networks, VANETs has its own unique characteristics, such as dynamic topology, high mobility and volatility, which render it vulnerable to various types of attacks from malicious vehicular nodes. Malicious nodes behave in different ways. For example, they may broadcast false information, which causes traffic jams or threatens drivers’ lives; they may intentionally drop a received message or refuse to help other vehicular nodes’ forward messages. Therefore, it has become an important yet practical problem to identify malicious nodes and detect forged messages in VANETs.

There exist many solutions in the literature, which can be divided into three categories. (i) Entity-oriented trust models, which evaluate the trustworthiness levels of vehicle nodes to identify selfish or malicious nodes [2,3]. (ii) Data-oriented trust models, which detect malicious nodes by evaluating the trustworthiness of messages [4]. (iii) Hybrid trust models, which combine both entity-oriented and data-oriented methods [5]. In addition, some security certification schemes have been proposed to determine the legitimacy of a vehicle node, such as the methods based on frequency identification [6], anonymous certificates [7,8] and group signature [9,10].

Blockchain is the underlying technology for the Bitcoin protocol that emerged in 2008 [5]. Blockchain provides a secure shared database as a ledger or log of transactions, without requiring a central trusted party for management. The consistency of blockchain is guaranteed through a distributed consensus protocol, where a set of participants (validators), in a trust-less, peer-to-peer network, collaborate in a completely transparent way to accept only valid transactions. These significant features of blockchain make it an excellent candidate for establishing a desirable trust model in VANETs. However, the original design goal of Bitcoin did not consider the privacy of nodes. By reviewing the ledger, the transactions made with any public key are traceable to a real identity.

Considering the characteristics of blockchain, we propose a security scheme to identify malicious nodes and detect forged messages based on the technology of blockchain while simultaneously preserving the identity privacy of vehicles.

In particular, compared with the existing methods, our work makes the following main contributions:

(1) We developed a blockchain-based security scheme for VANETs, where the blockchain on RSUs is able to identify malicious nodes and detect forged messages, and the blockchain on CAs is able to issue and revoke certificates for vehicles.
(2) We designed an incentive consensus mechanism on RSUs to encourage cooperative behaviors using award and punishment measures.
(3) Combined with the storage characteristics of blockchain, we designed a public–private key pseudonym strategy to protect the privacy of vehicles.

The rest of this paper is organized as follows. In Section 2, we summarize the related work. In Section 3, we formulate the problem under study. Section 4 details the proposed scheme. Section 5 presents and analyzes the simulation results. We conclude our work in Section 6.

## 2. Related Work

Differently from mobile ad hoc networks (MANETs), VANETs have several unique characteristics which make it challenging to design an effective scheme to identify malicious nodes and detect forged messages. First of all, its characteristic of high mobility makes it unpractical to maintain long-term interactions between vehicular nodes. Secondly, the topology of VANETs is subject to constant, rapid changes. Thirdly, application scenarios of VANETs are complicated and varying over time. A desirable scheme should function efficiently at any level of traffic density and preserve the privacy of vehicular nodes simultaneously.

In this section, we present a survey of the existing methods for identifying malicious nodes in VANETs and discuss the applications of blockchain in VANETs.

### 2.1. Detection Models in VANETs

The detection methods in VANETs fall into three categories. The first category is based on entity-oriented trust models to identify malicious nodes and permanently or temporarily prevent them from transmitting or forwarding any information.

Gong et al. [11] proposed a social-contribution-based routing protocol for vehicular networks with selfish nodes. The protocol considers two factors when making a forwarding decision, namely, the probability of delivery to the destination and the social contribution of the relay node. A node with a low social contribution and a high probability of delivery is preferred as the next hop node. Sedjelmaci et al. [12] proposed an efficient and lightweight intrusion detection mechanism in vehicular networks. This mechanism can not only detect internal and external attacks, but also defend against denial of service (DoS) attacks, integrity targets, and false alarm attacks. Compared with the contemporary detection schemes, it only uses cryptography algorithms to protect vehicular networks from external attacks.

Further efforts have been made over years in this direction. Khan et al. [13] proposed a new scheme named DMN for detecting malicious nodes in VANETs. The proposed scheme is node-centric and uses a monitoring approach to identify and isolate malicious nodes. DMN is an optimized DMV algorithm and considers multiple parameters to select a certain node as the verifier node. Haddadou et al. [14] proposed a distributed VANETs trust model named DTM2 and used a Markov chain for modeling. In this model, malicious nodes can be detected and evicted by a self-selection algorithm among network nodes. Moreover, the cooperative level of selfish nodes is improved by giving rewards to those nodes that enact active and cooperative behaviors. Bali et al. [15] proposed a novel secure clustering scheme for efficient data dissemination in VANETs. In order to calculate the trust between different devices, a trust metric is proposed based on the dynamic transmission characteristics of vehicles. On the basis of this trust metric, the proposed scheme designs secure clustering and trust establishment.

In entity-oriented trust methods, excluding malicious nodes from any operation may lead to disconnection and other issues. Many researchers believe that constructing trust in data is practically more useful than reporting on their nodes. Data are the foundation of applications in VANETs. Sharing trustworthy, safe and efficient data are critical to the performance of transportation. Therefore, the second category of methods focus on identifying the trustworthiness of data.

Shaikh et al. [16] proposed a decentralized trust management scheme for vehicular ad-hoc networks based on identity and anonymity, which can detect false location and false timestamp information. Gurung et al. [17] proposed a new trust model that can directly evaluate the credibility of message contents received from other vehicles. Various factors, such as content similarity, content conflict and route similarity, are considered to construct the trust model. Mohamed et al. [18] proposed a new voting-based enhancement algorithm (EVA) to improve the security of DSRC applications. The proposed algorithm reduces the time for decision making and increases the reliability of applications, but increases the delay and computational overhead of OBU. Alturkostan et al. [19] used a threshold-based method to check the security of messages and analyzed the impact of jamming attacks on the security scheme. On this basis, they proposed a new adaptive threshold algorithm that can effectively resist jamming.

The third category, defined as hybrid trust models, aim to ensure reliable communication between nodes and prevent malicious nodes from interfering with them. Therefore, the main goals of these models are to maintain communications, revoke suspicious nodes and stop malicious messages.

Saneeha et al. [20] proposed a trust model to evaluate the reliability of recommendations in VANETs. The trust model can effectively alleviate recommendation attacks and help nodes to identify malicious senders and incorrect recommendations. Yao et al. [21] proposed an entity-centered dynamic trust model to adapt to the dynamic environment in VANETs. In order to balance direct trust and recommendation, a dynamic adjustment factor α is introduced. The proposed trust model can evaluate the reliability of data. Li et al. [22] proposed an anti-attack trust management scheme (ART) for VANETs, which can detect and mitigate malicious attacks. It also considers the reliability of data and vehicle nodes in VANETs. Moreover, this trust scheme is not only suitable for a wide range of VANETs applications, but also can improve traffic efficiency. Ahmed et al. [23] proposed the notion of logistic trust to detect misbehavior in VANETs. In this scheme, the receiver uses the suggestions received from other nodes as its observation results, and then identifies the correctness of the information according to its own observation results and the trustworthiness of the sender. Sedjelmac et al. [24] proposed an accurate, lightweight framework for intrusion detection named AECFA, which is an improved algorithm based on security clustering and can detect dangerous attacks in VANETs. Sanjay et al. [25] proposed VSRP for communication between vehicles based on reputation evaluation systems. It is a stable and efficient method to detect malicious nodes in VANETs.

### 2.2. Applications of Blockchain in Vehicle Networking

Blockchain technology is an emerging distributed storage technology. The decentralized consensus mechanism used in blockchain effectively improves the security and privacy of the system, and brings considerable convenience to data exchange between connected smart devices. It has found many applications in various fields, especially in vehicular networks, for various purposes, such as data storage, identity authentication, privacy protection, and security trust. We summarize some of the typical security models based on blockchain technology in vehicular networks as follows.

Arora et al. [26] proposed an authentication and secure data transmission algorithm for Internet of Vehicles (IoV) using blockchain technology. This method can manage and calculate the trust of nodes in IoV and ensure secure communications between nodes. Lu et al. [27] proposed a blockchain-based anonymous reputation system (BARS) and established a privacy protection trust model in VANETs. To improve vehicle safety, it also integrates a reputation evaluation algorithm that relies on direct historical interactions and indirect opinions about vehicles. Yang et al. [28] proposed a decentralized trust management system for vehicular networks using blockchain technology. This method calculates the trust value based on Bayesian inference model to perform message verification, and all RSUs maintain and update the trust blockchain. Yang et al. [29] proposed a blockchain-based traffic event verification framework (BTEV) to complete event verification and alarm vehicle nodes near RSUs, and introduced a proof-of-event consensus mechanism (PoE), which can identify malicious behaviors and prevent the spread of false warning information. Wagner et al. [30] proposed a blockchain-based VANET framework which changes the transaction verification mechanism and management process of blockchain, and adds a trusted CA. This framework can realize the verification of traffic events without the assistance of RSUs and other infrastructures.

Malikl et al. [31] proposed a blockchain-based authentication and fast revocation framework for VANETs, which preserves vehicle nodes’ anonymity without revealing their real identities, and reduces the dependence on authentication and the computing, and the communication overhead. In addition, it also realizes the rapid updating of the status of revoked vehicles in the blockchain shared ledger. Lu et al. [32] proposed a blockchain-based anonymous reputation system (BARS), which uses two blockchains (CerBC and RevBC) to achieve authentication and revocation transparency, and designed a reputation management algorithm based on historical interaction and indirect opinion to prevent the spread of false messages. In addition, public keys are used as communication pseudonyms to protect the privacy of vehicle nodes. Khan et al. [33] proposed a blockchain-based security architecture that can prevent attacks in VANETs, such as denial of service (DoS) attacks, Sybil attacks, impersonation attacks, and replay attacks. This work designed a message rating and trust method based on blockchain to ensure the security and privacy of vehicle nodes. Li et al. [34] proposed a distributed architecture-based blockchain for VANETs, which has advantages in identity and privacy protection. The proposed architecture includes blockchain setup, registration of vehicles, SBMs upload, and blockchain record. It can effectively solve the problem of trust between entities and centralized deployment. Javaid et al. [35] proposed a distributed trust management scheme based on blockchain to achieve secure message sharing and privacy protection between vehicles in VANETs. It assigns a unique encrypted fingerprint to each vehicle, and a CA is used to eliminate the linkage between public key and real identity to protect the identities of vehicle nodes from attacks. Dai et al. [36] proposed a security framework based on reinforcement learning and blockchain, which firstly uses the blockchain to protect messages from tampering and records the trust of vehicle nodes on the blockchain, and then selects reliable intermediate nodes by using reinforcement learning.

In summary, the existing methods have been proven to be effective and successful in their targeted applications, but still face some technical challenges, including improving the accuracy of detection and enhancing protection of privacy. Therefore, we propose a security scheme based on blockchain technology to identify malicious nodes and detect forged messages. Meanwhile, we address the issue of privacy protection for vehicles and designed an incentive mechanism to encourage cooperative behaviors of RSUs. Additionally, the proposed security scheme enables decentralized management in vehicle networks and effectively reduces network overheads.

## 3. Problem Statement

In this section, we present application scenarios in VANETs, construct a model of VANETs based on blockchain, and define the objectives of our design.

### 3.1. Application Scenarios in VANETs

Generally, there are two types of communications in VANETs: vehicle to vehicle (V2V) and vehicle to infrastructures (V2I). Vehicle nodes equipped with sensors communicate with each other. They are able to send, transmit, and share various types of data, such as traffic condition, service information, and entertainment information. Vehicle nodes are also able to communicate with RSUs. Sharing information, especially traffic safety-related messages, is fundamental for vehicles in VANETs.

**Figure 1:**
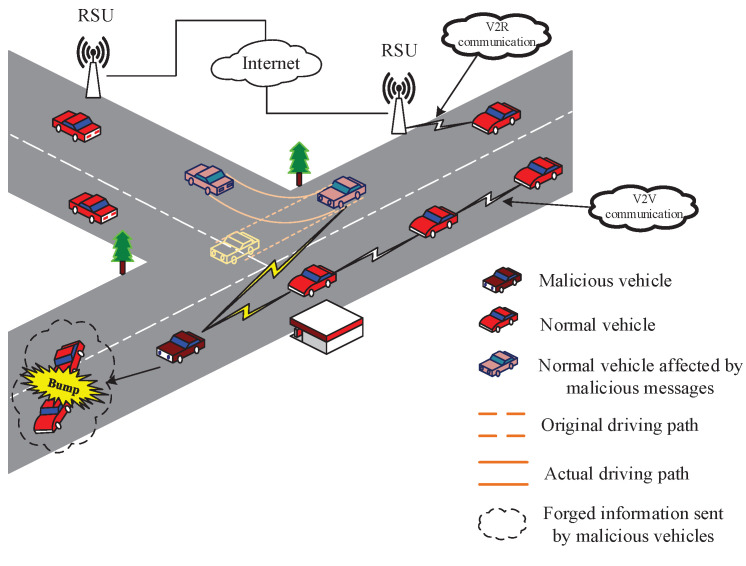
A scenario with malicious nodes in vehicular networks.

However, malicious vehicle nodes in VANETs may send forged messages for their own interests. In Figure 1, a malicious vehicle node sends a forged message that reports a crash accident ahead to its neighbor nodes. When a normal node receives the forged message, it will modify its original driving route accordingly. Such false information may affect the drivers’ judgment and endanger the safety of driving. Therefore, identifying forged messages and malicious vehicle nodes is the basis of secure communication in VANETs.

In this paper, we propose a security scheme based on blockchain to identify forged safety information (such as inclement weather broadcast, icy road, traffic jam, and traffic accident) and malicious vehicle nodes.

### 3.2. Blockchain-Based VANETs Model

**Figure 2:**
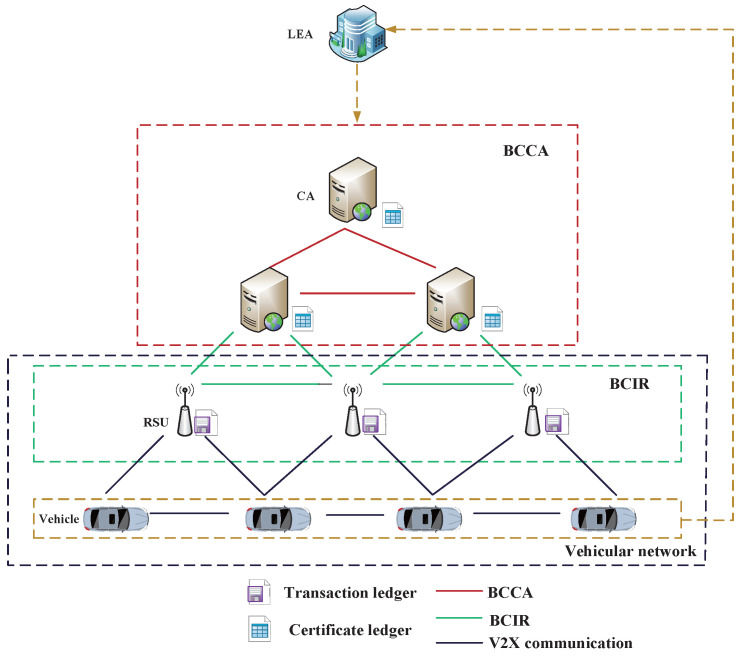
Blockchain-based vehicle networks.

We propose to adopt the technology of blockchain in VANETs, as illustrated in Figure 2, where there are four types of entities: vehicle nodes, RSUs, Certificate Authority, and Law Enforcement Authority, as described below.

- Law Enforcement Authority (LEA): The functions of LEA include registration of vehicles, authorization of a CA to issue certificates, etc.
- Certificate Authority (CA): A CA issues a certificate for a vehicle when it obtains a warrant from the LEA. All actions of a CA will be recorded transparently in the blockchain of CAs and can be verified by every CA in the VANETs.
- RSU: All broadcasted messages and transactions are verified by RSUs and then recorded in the RSU blockchain. The main functions of RSU include monitoring behaviors of vehicles and evaluating the reputation scores of each vehicle.
- Vehicles: Vehicles equipped with OBU devices are moving entities and communicate with each other to share various types of messages.

There are two blockchains in the proposed scheme:

(1) Blockchain for Certification on CA (BCCA)

BCCA acts as the public ledger for all issued certifications. All actions of a CA are recorded transparently in the BCCA. A transaction in the BCCA refers to a message broadcasted by a CA to issue or revoke a certificate. Each transaction contains the timestamp and the digital signature of the CA. In order to preserve the privacy of vehicles, no information linkable to the real identity is included in the transaction.

(2) Blockchain for Identification on RSU (BCIR)

BCIR acts as the public ledger for all transactions, which include identifying forged messages or malicious vehicle nodes among RSUs. All actions of a RSU are recorded transparently in the BCIR blockchain. A transaction in BCIR refers to a message broadcasted by a RSU, which is able to identify the trustworthiness of message sharing in VANETs, and also evaluate the behaviors of vehicles. The results of identification or evaluation are broadcasted and regarded as transactions.

BCCA and BCIR bring considerable convenience to data exchange between connected smart device, such as CAs or RSUs. In BCCA, the blockchain technology is able to secure assigning and revoking certifications for vehicles. In BCIR, the blockchain technology is able to secure management of vehicles’ reputations and ensure secure communications between RUSs. Simultaneously, it also can help identify malicious nodes and detect forged messages.

Each vehicle registers with the LEA using its own real identity to ensure the traceability of malicious nodes. The CA assigns a certification to a vehicle if it achieves a warrant from the LEA. Certifications help vehicles construct trusted relationships when a vehicle communicates with other vehicles or RSUs.

**Figure 3:**
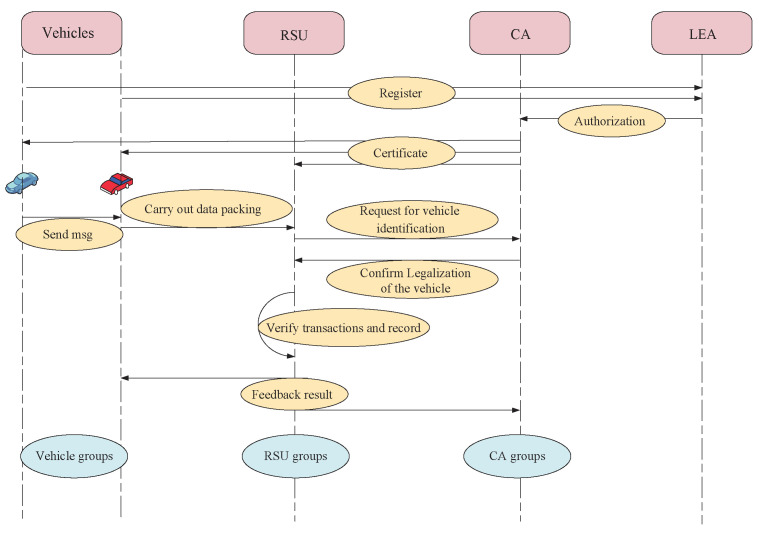
The flow chart of detection.

In the framework illustrated in Figure 3, node $V_{j}$ receives a message sent by $V_{i}$. To assess the trustworthiness of the message, $V_{j}$ sends this message to the nearest RSU. To determine the legitimacy of $V_{j}$, the RSU sends $V_{j}$’s pseudonym to the nearest CA when it receives the message from $V_{j}$. As the blockchain of CA contains the linkages between the vehicles’ pseudonyms and their real identities, the CA is able to identify whether or not $V_{j}$ is a legitimate node and sends the result of identification to the RSU. If $V_{j}$ is not a legitimate node, the RSU drops this message; otherwise, the blockchain of RSU evaluates the trustworthiness of the message and sends the result of evaluation to $V_{j}$.

### 3.3. Design Objectives

Due to the unique characteristics of high mobility and limited connectivity, VANETs are prone to various types of cyber attacks from malicious vehicle nodes, which affect the efficiency of VANETs and threaten the safety of drivers. Towards this end, we propose a detection scheme based on a blockchain consensus mechanism following several design goals:

- The first goal was to address the problem of centralization in vehicular networks. Our work adopted blockchain technology to construct a novel decentralized architecture for vehicular networks.
- The second goal was to design a malicious node identification scheme based on an incentive consensus mechanism which is able to identify the legitimacy of a received message and detect malicious nodes by evaluating the trustworthiness of the source nodes. Meanwhile, the consensus is able to stimulate RSUs to enact active behaviors.

## 4. A Security Scheme Based on a Blockchain Consensus Mechanism

In this section we propose a blockchain-based security method, referred to as *BCSM*, and present its design details.

### 4.1. Assumption

Firstly, we give some necessary assumptions as the foundation of the proposed scheme.

(1) The adversary is not able to compromise more than a half of the vehicles in the network. This is a reasonable assumption in practice.
(2) Certification Authorities (CAs) and RSUs are equipped with customized hardware with high computing power.
(3) Cryptography technology provides a secure communication channel between entities as long as the secret key is not compromised.

### 4.2. Initialization of VANETs Based on Blockchain

Initially, each entity on BCCA and BCIR generates a pair of private and public keys. When a vehicle $V_{i}$ enters VANETs, it sends a message that contains its private information to prove its legitimate identification. If the message is valid, LEA sends a signed warrant to the CA, which then issues an initial certificate to vehicle $V_{i}$. LEA stores the received message in the database with high-level security, which will be used for tracking the vehicle’s real identity in cases of disputes.

(1) Blockchain establishment: There are two blockchains set up during this stage. All CAs form a blockchain for certification, and all RSUs form a blockchain for identification. Each member of a blockchain is equal and has the same rights and obligations.
(2) Certification: $V_{i}$ registers with a CA for the first time by submitting its vehicle ID obtained from the LEA. The CA verifies the vehicle ID and issues a certification to $V_{i}$, which contains the expiration date, a pseudo-ID $PID_{V_{i}}$, the public–private keys, the signatures of authorities, and the initial reputation value assigned for $V_{i}$.

The blockchain of CA maintains a database, which stores the hash map of a mapping of pseudo-IDs of vehicles with the certificates. This blockchain ensures the traceability of issuing or revoking certificates.

### 4.3. Detection Scheme

Messages transmitted in VANETs are described in Table 1 and fall into three categories: beacon messages, alert messages, and entertainment messages. Alert messages are broadcast in an emergency and are critical to safe driving. Therefore, in this paper, we focus on how to detect forged alert messages based on the blockchain technology in VANETs. Alert messages normally report emergency situations described in Table 2, where $TTL_{event}$ and $Ran_{msg}$ denote life cycle and the longest transmission range of the event reported in an alert message, respectively. As shown in Table 2, alert messages are divided into four categories and have different life cycles.

**Figure 4:**
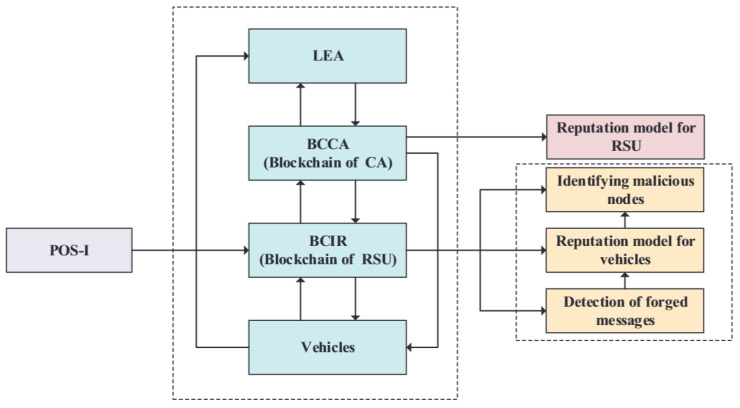
The scheme structure.

**Figure 5:**
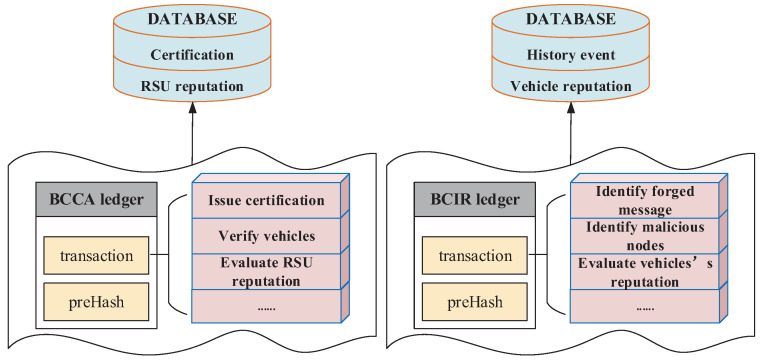
The ledgers of BCIR and BCCA.

As illustrated in Figure 4, the security scheme is comprised of four components: identification of the legitimacy of a vehicle; an incentive consensus mechanism; identification of forged messages and malicious vehicle nodes; and the public–private key mechanism and RSA encryption algorithm are adopted in the scheme to protect the security and privacy of vehicles. The ledgers of BCIR and BCCA in this scheme are illustrated in Figure 5.

(1) **Verify a vehicle’s legal identification**

When $V_{i}$ receives an alert message from $V_{j}$, it sends the received message to a nearby RSU and requests it to identify the legitimacy of the message. The RSU checks if $V_{i}$ has a legal identification before evaluating the trustworthiness of the message. Algorithm 1 illustrates the steps to verify the identification of $V_{i}$. For secure communication, the RSA method is adopted in Algorithm 1.

Step 1: Vehicle $V_{i}$ sends a request to a RSU within its communication range to verify the message received from vehicle $V_{j}$. The request includes the received message, the pseudo-ID $PID_{V_{i}}$, and $PK_{V_{i}}$, which is the public key of $V_{i}$.

Step 2: After receiving the request, the RSU sends a message, which includes $PID_{V_{i}}$ and a random value *L* to the closest CA. Note that *L* is produced by the method of linear congruence generator (LCG), calculated as:

(1) $$\left\{\begin{array}{c} L_{0}=d\\ L_{r+1}=\left(A*L_{r}+Z\right)\;\mathrm{mod}\;\left(M\right) \end{array}\right.,$$

where *d* is a seed value, and its initial value is set to be the current system time; *A* is a multiplier; *Z* is an increment value; and *M* is a modulus. Note that *M* and *Z* are prime numbers to each other.

Step 3: When the CA receives the message from the RSU, it checks the ledger of BCCA. If $PID_{V_{i}}$ is stored in the ledger, then $V_{i}$ is a legitimate vehicle; otherwise, it is an illegitimate one. Then, a session key $k_{s}$ is created by the random value *L* encrypted with the private key of the CA. $E_{PB_{RUS}}\left[k_{s}\right]$ is created by encrypting $k_{s}$ with the public key of the RSU $PB_{RSU}$ and $E_{k_{s}}\left[report\right]$ is created by encrypting the checking report with $k_{s}$. Finally, $E_{k_{s}}\left[report\right]$ and $E_{PB_{RUS}}\left[k_{s}\right]$ are encrypted with $PB_{RSU}$ and sent to the RSU. The encryption is performed as

(2) $$E:C=E_{PB_{RSU}}\left[E_{PB_{RSU}}\left[K_{s}\right]E_{K_{s}}\left[report\right]\right],$$

where *E* is a function of encryption and *C* represents the plaintext.

Step 4: When the RSU receives the encrypted result from the CA, it decrypts the result with its own $PK_{RSU}$ to obtain $E_{k_{s}}\left[report\right]$ and $E_{PB_{RUS}}\left[k_{s}\right]$. Then, the RSU decrypts $E_{PB_{RUS}}\left[k_{s}\right]$ with its $PK_{RSU}$ to obtain $k_{s}$. Finally, the report is created by decrypting $E_{k_{s}}\left[report\right]$ with $k_{s}$. This decryption process is described as

(3) $$D:\left[E_{PB_{RSU}}\left[K_{s}\right]E_{K_{s}}\left[report\right]\right]=D_{PB_{RSU}}\left[C\right],$$

where *D* is a function of decryption.
**Algorithm 1** Verify a vehicle’s legal identification**Input:** 
$V_{i}$, $PIDV_{i}$, $PB_{RSU}$ and $PK_{CA}$ ;**Output** 
verification result; 1:$V_{i}$ sends a request to BCIR; 2:LCG is used to produce *L*; 3:*L* and $PIDV_{i}$ are sent to BCCA; 4:CA checks the ledger on BCCA; 5:**if**$PIDV_{i}$ is stored in the ledger **then** 6:     $V_{i}$ is a legal vehicle; 7:**else** 8:    $V_{i}$ is an illegal vehicle; 9:**end if** 10:Encrypt *L* with $PK_{CA}$ as $K_{s}$; 11:Encrypt the verification result with $K_{s}$ as $E_{K_{s}}\left[report\right]$; 12:Encrypt $E_{K_{s}}\left[report\right]$ and $E_{PB_{RSU}}\left[K_{s}\right]$ with $PB_{RSU}$; 13:Send $E_{PB_{RSU}}\left[K_{s}\right]\left|\right|E_{K_{s}}\left[report\right]$ to the RSU; 14:The RSU decrypts $E_{PB_{RSU}}\left[K_{s}\right]\left|\right|E_{K_{s}}\left[report\right]$; 15:Output the verification result.

(2) **POS consensus with an incentive mechanism**

If $V_{i}$ is verified as a legitimate vehicle by the BCCA blockchain, the BCIR blockchain then identifies whether or not the message sent by $V_{i}$ was forged.

To stimulate the RSUs to take active behaviors, a consensus appropriate for VANETs should be constructed. Consensus in a blockchain is a process where all peers of the network reach a common agreement on the present state of the distributed ledger. At present, the most common consensus algorithms include POW, POS, and PBFT. From the advent of Bitcoin to today, there over 30 consensus algorithms have emerged [37], most of which are based on the above three consensus algorithms.

Unlike other traditional consensus, nodes in BCIR are designed to utilize computing power for forged message validation rather than merely solving the difficult hash problem. Therefore, we designed a novel consensus POS based on an incentive mechanism (POS-I) for BCIR in VANETs. According to the POS consensus with an incentive mechanism, when an RSU enacts active behavior, it receives energy benefit. POS-I is described in Algorithm 2.
**Algorithm 2** Consensus mechanism POS-I**Input:** 
$Egy_{o}$, *k*, $Th_{Egy}$, $\Delta T_{\mathrm{pos}}$, *a*, *J*, Pr;**Output** 
committer peer; 1:RSU sends a request to CA; 2:BCCA initializes an election for selecting committer peer; 3:**for all** RSU participating in the election **do** 4:   $R_{l}$ submits $\Delta Egy_{consume\_l}=\frac{k}{2(k+1)}Egy_{o\_l}$ as deposit; 5:   Calculate $Egy_{e\_l}=Egy_{o\_l}-\Delta Egy_{consume\_l}$; 6:    **if**  $Egy_{e\_l}<Th_{Egy}$ **then** 7:         $R_{l}$ cannot participate in the election; 8:    **else** 9:         $R_{l}$ is regarded as a candidate; 10:   **end if** 11:    Calculate $Stake_{R-l}=\sum_{x=1}^{J}Egy_{x}*{\left(1+a\%\right)}^{J}$; 12:**end for** 13:Selecting the node whose has $Max_{stake}$ as the committer peer ; 14:Calculate $\Delta Egy_{reword}=\frac{1-Egy_{J-1}}{2}$; 15:Calculate $Egy_{J}=Egy_{J-1}\times e^{\frac{1}{\Delta T_{J-1\_J}}}+\Delta Egy_{consume}\times Pr+\Delta Egy_{reword}$; 16:Output committer peer.

Step 1: BCCA initializes an election to select a committer peer. The RSUs, which would like to participate in the election, submit deposits in order to become candidates. The energy value of every candidate RSU is reduced as a deposit. The process of calculating deposit is described as:

(4) $$\Delta Egy_{consume\_l}=\frac{k}{2(k+1)}Egy_{o\_l},$$

where $\Delta Egy_{consume\_l}$ denotes the submitted deposit, $Egy_{o\_l}$ denotes the current energy value of $RSU_{l}$, *k* denotes the total number of participation elections of $RSU_{l}$, and the initial value *k* is set to zero.

RSUs have various types of behaviors in VANETs, such as broadcasting messages, participating election of selecting a committer peer, and identifying the trustworthiness of a message. Normally, the energy of a RSU changes with different behaviors. For example, when it broadcasts messages in VNAETs, its energy is consumed. Meanwhile, in order to encourage its active behaviors, it is also rewarded a certain energy. The reward is larger than the consumed energy.

After submitting the deposit, the energy of $R_{l}$ is updated as:

(5) $$Egy_{e\_l}=Egy_{o\_l}-\Delta Egy_{consume\_l}.$$

Step 2: If the energy of $R_{l}$ is lower than $Th_{Egy}$, which is a threshold, $R_{l}$ is deleted from the candidate group; otherwise, it remains in the candidates group.

Step 3: The total number of elections initialized by BCCA is counted as *J*. After every election for a committer, the energy of every RSU on BCIR is updated. Stakes refer to the assets (or energy) owned by a node. The idea is that the more active behaviors an RSU has, the more stakes it owns. The stake of each RSU candidate is calculated as

(6) $$Stake_{R-l}=\sum_{x=1}^{J}Egy_{x}*{\left(1+a\%\right)}^{J},$$

where $Stake_{R-l}$ denotes the stake of $R_{l}$, and $Egy_{x}$ denotes the energy value of $R_{l}$ after the *x*-th election. The RSU, which has the highest stake value among the candidate group, is selected as the committer peer.

Step 4: When the committer peer is selected according to the above process, BCCA refunds a certain percent of deposit to each candidate RSU. Now, BCCA updates all RSUs. The updating process is described as

(7) $$Egy_{J}=Egy_{J-1}\times e^{\frac{1}{\Delta T_{J-1\_J}}}+\Delta Egy_{consume}\times Pr+\Delta Egy_{reword},$$

where $Egy{}_{J}$ denotes the energy of a RSU after the Jth election initialized by BCCA, $Egy_{J-1}$ denotes the energy of a RSU after the (J−1)th election, and $P_{r}$ denotes a certain percent of deposit refunded. Especially, we set $e^{\frac{1}{\Delta T_{J-1\_J}}}$ to be an attenuation coefficient. Meanwhile, BCCA offers a reward $\Delta Egy_{reword}$ to the committer peer, calculated as

(8) $$\Delta Egy_{reword}=\frac{1-Egy_{J-1}}{2}.$$

As mentioned above, if $RSU_{l}$ is selected as a committer peer on BCIR, it gains the reward energy and be refunded at a certain percentage.

(3) **Verify the integrity of messages**

The RSU, which is elected as a committer peer, verifies the message integrity sent from $V_{i}$. Algorithm 3 describes the verification process for message integrity.
**Algorithm 3** Verify message integrity**Input:** 
$PK_{committer}$, $PB_{committer}$ , $PK_{CA}$, $PB_{CA}$;**Output** 
Message integrity result; 1:Calculate the hash code h(m) of *m*; 2:Encrypt h(m) with the $PK_{committer}$ as the committer $RSU{}^{\prime }s$ digital signature; 3:Add the digital signature in the message *m* to get $m{}^{\prime }$; 4:Use LCG to get a random number *G*; 5:Encrypt G with the $PB_{committer}$ as $Committer_{K_{s}}$; 6:Encrypt $m{}^{\prime }$ with $Committer_{K_{s}}$ as $E_{Committer_{K_{s}}}\left[m{}^{\prime }\right]$; 7:Encrypt $Committer_{K_{s}}$ with $PB_{CA}$ as $E_{PB_{CA}}\left[Committer_{K_{s}}\right]$; 8:Send $E_{Committer_{K_{s}}}\left[m{}^{\prime }\right]$ and $E_{PB_{CA}}\left[Committer_{K_{s}}\right]$ to BCCA; 9:CA decrypts $E_{PB_{CA}}\left[Committer_{K_{s}}\right]$ with $PK_{CA}$ to get $Committer_{K_{s}}$; 10:Decrypt $E_{Committer_{K_{s}}}\left[m{}^{\prime }\right]$ with $Committer_{K_{s}}$ to get digital signature; 11:Decrypt digital signature with $PB_{committer}$ to get h(m); 12:CA on the message *m* hashes to get H(m); 13:**if** h(m)=H(m)**then** 14:    the *m* is verified as integrity; 15:**else** 16:    the *m* is tampered; 17:**end if** 18:Output the message integrity result.

Step 1: The committer RSU on a message *m* hashes to get the synopsis of *m*, which is denoted as h(m). Then, h(m) is encrypted with the committer RSU’s private key $PK_{committer}$. The result of encryption is considered as the committer RSU’s digital signature and is added in the message *m* to get $m^{\prime }$.

Step 2: The committer RSU employs the LCG method to obtain a random number *G*, and a session key $Committer_{K_{s}}$ is created by encrypting *G* with the committer RSU’s public key $PB_{committer}$.

Step 3: $E_{Committer_{K_{s}}}\left[m^{\prime }\right]$, which is created by encrypting $m^{\prime }$ with $Committer_{K_{s}}$, and $E_{PB_{CA}}\left[Committer_{K_{s}}\right]$ are sent to the CA, which is the closest to the RSU.

Step 4: The CA decrypts $E_{PB_{CA}}\left[Committer_{K_{s}}\right]$ with its private key $PK_{CA}$ to get $Committer_{K_{s}}$, and $E_{Committer_{K_{s}}}\left[m^{\prime }\right]$ is decrypted with $Committer_{K_{s}}$ to get *m* and the committer RSU’s digital signature.

Step 5: The committer RSU’s digital signature is decrypted with $PB_{Committer}$ to get h(m). The CA that is the closest to the RSU on the message *m* hashes to get H(m). If h(m) is equal to H(m), *m* is verified as integrity.

(4) **Verify the legitimacy of messages.**

In our scheme, BCIR acts as a distributed public ledger, which stores the reputation values of every vehicle in the blockchain. BCIR first checks the sender vehicle $V_{j}$’s reputation and then verifies the trustworthiness of the message. If the reputation of $V_{j}$ is smaller than the reputation threshold $R_{threshold}$, the message from this node is labeled as forged information. Otherwise, BCIR checks the message based on the evidence with respect to EventType, $Loc_{event}$, EventTime, time effectiveness, and distance effectiveness.

As mentioned above, the committer RSU follows the message verification policies to determine the message’s trustworthiness as follows:

- Check the sender’s reputation from the vehicle reputation table on BCIR.
- Check EventType, $Loc_{event}$, and EventTime.
- Check time effectiveness and distance effectiveness.

If the sender’s reputation is larger than $R_{threshold}$, EventType, $Loc_{event}$, and EventTime of the received message are checked in the historical event table on BCIR in order to evaluate if an event reported in the message has been stored in the historical event table.

If EventType of a message in the historical event table is the same as $m_{EventType}$, the message is placed in $S_{m}$, defined as $S_{m}=\left\{e_{1},e_{2},...,e_{z}\right\}$, where *z* denotes the total number of messages in $S_{m}$. For $e_{q}\in S_{m}$, the distance between $e_{q}$ and *m* is calculated as:

(9) $$Dis=\sqrt{{\left({m_{x}}_{Loc_{event}}-e_{q}{{}_{x}}_{{}_{Loc_{event}}}\right)}^{2}+{\left({m_{y}}_{Loc_{event}}-e_{q}{{}_{y}}_{{}_{Loc_{event}}}\right)}^{2}+{\left(m_{EventType}-e_{q_{EventType}}\right)}^{2}},$$

where $m_{Loc_{event}}=<m_{xLoc_{event}},m_{yLoc_{event}}>$, where $m_{xLoc_{event}}$ and $m_{yLoc_{event}}$ denote the longitude and latitude of *m*, respectively; and $e_{q_{Loc_{event}}}$ is defined as $<e_{qx_{Loc_{event}}},e_{qy_{Loc_{event}}}>$, which denote the longitude and latitude of $e_{q}$, respectively. The similarity of $e_{q}$ and *m* is calculated as

(10) $$Sim_{e_{q}\_m}=\frac{1}{1+Dis}.$$

If $Sim_{e_{q}\_m}$ is larger than $Th_{Sim}$, *m* is considered to be the same as $e_{q}$, and it checks time effectiveness and distance effectiveness. Otherwise, *m* is treated as a new one.

If Δt is over $Th_{t}$, the distance effectiveness is checked; otherwise, it is dropped because it expires:

(11) $$\left\{\begin{array}{c} \Delta t<Th_{t},\\ \Delta t=RecTime-EventTime. \end{array}\right.$$

If Δt is over $Th_{d}$, the message is dropped; otherwise, it is considered to be a reliable message:

(12) $$\left\{\begin{array}{c} \Delta d<Th_{d},\\ \Delta d=Loc_{vehicle}-Loc_{event}. \end{array}\right.$$

The process for identifying the trustworthiness of a message is illustrated in Algorithm 4.
**Algorithm 4** Verify the legitimacy of messages**Input:** 
$R_{threshold}$, $Th_{Sim}$, $Th_{t}$, $Th_{d}$;**Output** 
the legitimacy of messages; 1:For a message *m*; 2:**if**$R_{source}<R_{threshold}$**then** 3:    *m* is verified as a forged message; 4:**end if** 5:**for all** events recorded in historical event table on BCIR **do** 6:    Compare EventType of the event with $m_{EventType}$; 7:    **if** EventType of the event is the same as $m_{EventType}$ **then** 8:        Place the event in $S_{m}$; 9:        **for all** events in $S_{m}$ **do** 10:           Calculate $Sim=\frac{1}{1+Dis}$, $e_{q}\in S_{m}$; 11:           **if** $Sim>Th_{Sim}$ **then** 12:               The event in *m* is the same as $e_{q}$ ; 13:           **end if** 14:        **end for** 15:    **else** 16:        Calculate Δt=RecTime−EventTime; 17:        **if** $\Delta t>Th_{t}$ **then** 18:           *m* is outdated and discarded; 19:        **else** 20:           Calculate $\Delta d=Loc_{vehicle}-Loc_{event}$; 21:           **if** $\Delta d<Th_{d}$ **then** 22:               *m* is a legitimate message; 23:           **end if** 24:        **end if** 25:    **end if** 26:**end for**

If the received message is valid and trustworthy based on the aforementioned policy, it is stored in the historical event table on BCIR and the sender vehicle’s reputation is increased. Otherwise, the sender vehicle’s reputation is decreased. When a vehicle’s reputation value is below the threshold $R_{V}$, it is considered to be a malicious node. The reputation of $V_{i}$ is calculated as

(13) $$R_{V_{j\_T}}=R_{V_{j\_T-1}}\times \frac{True_{msg\_T}}{Sum_{msg\_T}},$$

where $R_{V_{j\_T}}$ represents the reputation value of $V_{j}$ in the time period *T*, $R_{V_{j\_T-1}}$ denotes $V_{j}$’s reputation in the time period T−1, and $Sum_{msg\_T}$ and $True_{msg\_T}$ represent the total numbers of messages and forged messages sent from $R_{V_{j}}$ in the time period *T*, respectively.

## 5. Simulation-Based Experiments and Performance Analysis

We present the performance evaluation of our proposed blockchain-based VANETs architecture in this section.

### 5.1. Simulation Environment

#### Simulation System Setup

To test and optimize the performance of the security scheme, we used ONE to simulate the network. Meanwhile, the SUMO (Simulation of Urban Mobility) framework was used as the mobility generator to prototype inter-modal traffic systems.

The vehicles in the SUMO simulator are shown as the dynamic nodes in the ONE framework. We programmed their functionality and behavior while the movement utilized the built-in libraries and procedures. In the simulation-based experiments, we used the shortest path map-based movement model in ONE to simulate vehicle behaviors on the road. The model initially places the nodes at random locations, but selects specific destinations for all nodes in the map and uses Dijkstra’s shortest path algorithm to find the shortest path to the destination.

**Figure 6:**
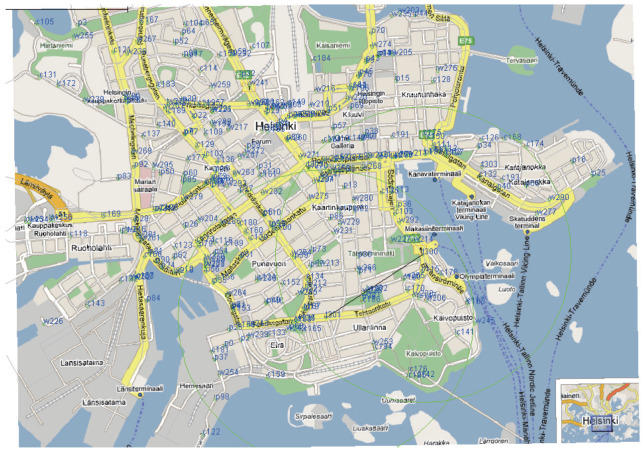
Helsinki City map (Finland).

There were two scenarios in the simulation experiments: low traffic density and high traffic density. As shown in Figure 6, the vehicle node performed simulated motion based on the Helsinki City map (Finland). The settings of the nodes are not arbitrary and include various types: emergency vehicles, such as police cars and ambulances; vehicles with fixed lines, such as buses and trams; and randomly distributed vehicles, such as private cars and taxis.

The specific simulation parameters are shown in Table 3. Each simulation is repeated twenty times to calculate the average. In our experiments, the number of malicious vehicles that sent forged or bogus messages in VANETs varied from 10% to 45%. When the malicious vehicles were over 45%, VANETs had drastically decreasing efficiency and could not provide any reliable services. Hence, we did not consider this extreme situation.

**Figure 7:**
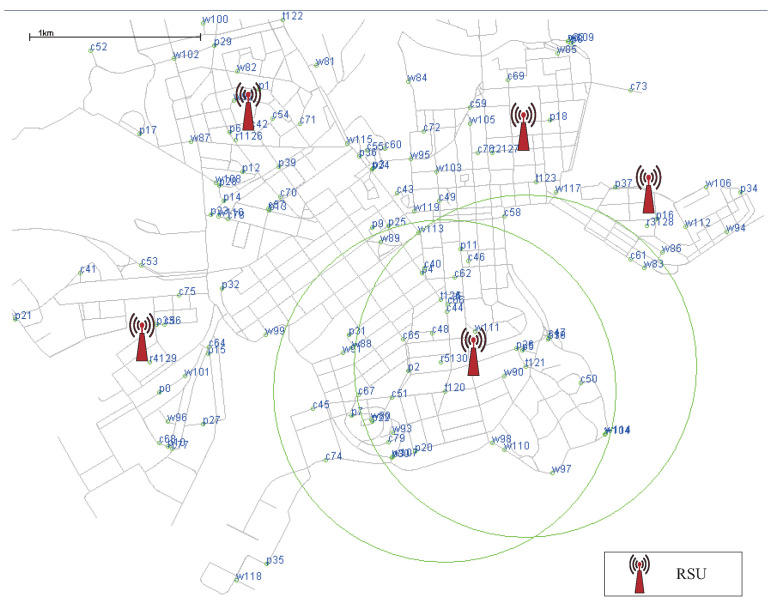
Locations of all nodes before the simulation started.

Additionally, several fixed nodes are manually added to the map to function as RSU infrastructure. The locations of all nodes are shown in Figure 7, where we also identify the specific locations of the deployed RSUs.

We conducted detailed analysis of the experiments under these parameters and simulation settings in the following section.

### 5.2. Simulation Results and Analysis

In order to evaluate the proposed scheme, we ran two sets of experiments: the first evaluated the detection of malicious forged messages; the second set evaluated the performance of the protocol combined with the security scheme.

#### 5.2.1. Evaluate the Detection of Forged Messages

To evaluate the detection performance of the proposed scheme, we considered false alarm rate (FAR) and missed detection rate (MDR) as the performance metrics:

(14) $$FAR=\frac{N_{f}}{N_{i}},$$

(15) $$MDR=\frac{N_{miss}}{N},$$

where $N_{f}$ denotes the total number of true messages that are identified as false messages by the method, $N_{i}$ denotes the total number of true messages, $N_{miss}$ denotes the total number of false messages that are identified as true messages by the method, and *N* denotes the total number of false messages.

We chose HCPDS [38] and GBPM [39] as baseline methods for comparison. BCSM is the security mechanism proposed in this paper. Note that there are two blockchains in BCSM: BCCA and BCIR. In our experiments, POS consensus with an incentive mechanism was used to encourage RSUs to take active behaviors. We compared BCSM with the baseline methods mentioned above.

**Figure 8:**
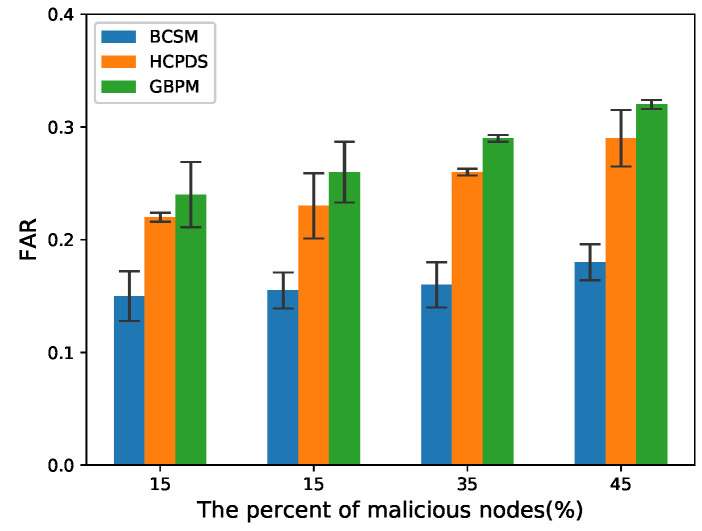
FAR for low traffic density.

**Figure 9:**
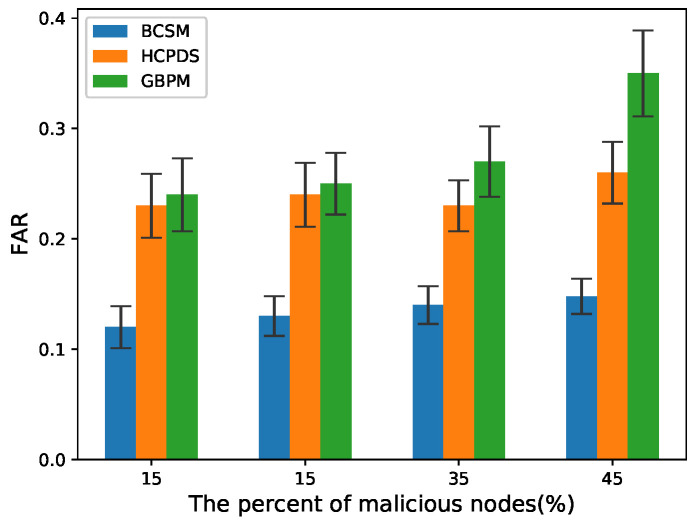
FAR for high traffic density.

Figure 8 and Figure 9 plot the FAR in high and low traffic density scenarios, respectively. Compared with HCPDS and GBPM, BCSM had lower FAR because it adopts two types of blockchain BCIR and BCCA to validate the legitimacy of sender nodes and identify the trustworthiness of messages transmitted in VANETs.

**Figure 10:**
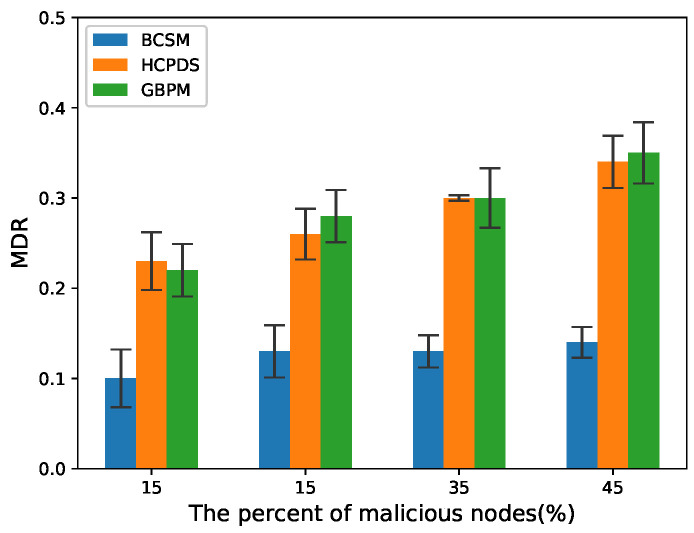
MDR for low traffic density.

**Figure 11:**
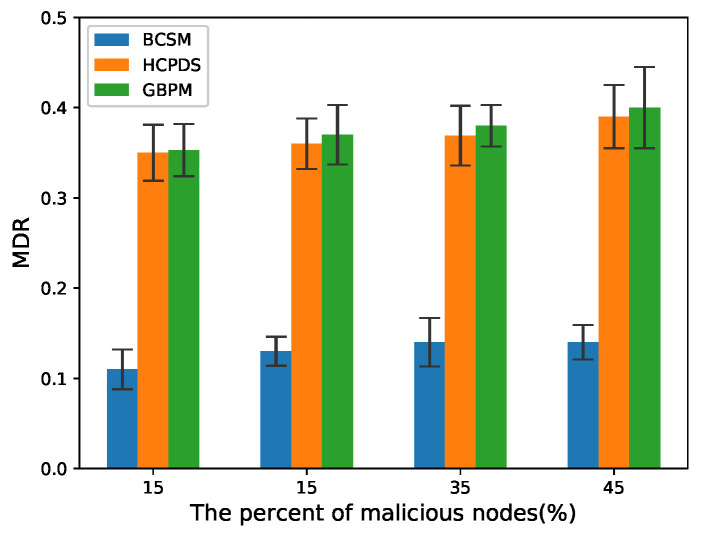
MDR for high traffic density.

Figure 10 and Figure 11 plot the MDR in high and low traffic density scenarios, respectively. As the percentage of malicious nodes increases from 15% to 45%, the MDR of HCPDS and GBPM increases. Compared with these two methods, BCSM is able to effectively mitigate the negative effect from the increase of malicious vehicle nodes and maintain a relatively stable level in terms of MDR.

#### 5.2.2. Evaluate the Performance of Transmission

For the evaluation of message transmission, we considered three performance metrics: delivery rate, delay and overhead rate.

(1) Delivery rate: It refers to the percentage of successful transmissions among all messages sent by nodes in the network. The higher the delivery rate, the higher the communication quality between nodes, and the better the overall network performance.
(2) Delay: It refers to the average time from creating a message to successfully delivering the message to the target node. The shorter the delay, the better the overall simulation performance.
(3) Overhead rate: It refers to the difference between the number of forwarded and the number of delivered messages.

We compare the proposed scheme with AODV, TBM [40], and BSIA [31], and summarize the simulation results as follows.

**Figure 12:**
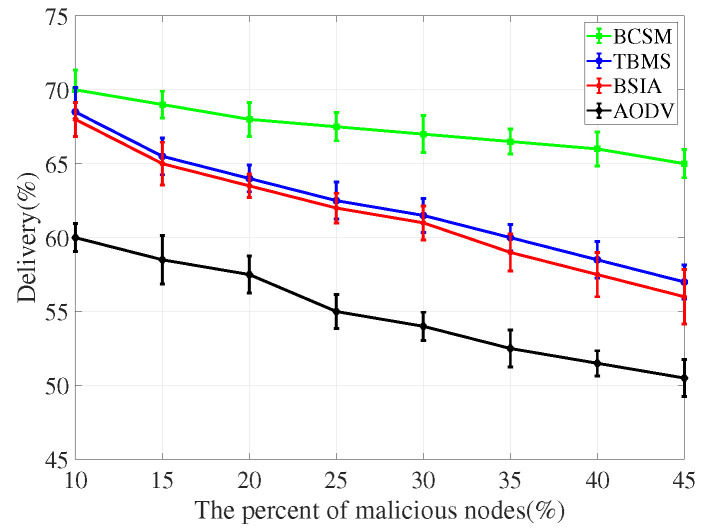
Delivery for low traffic density.

**Figure 13:**
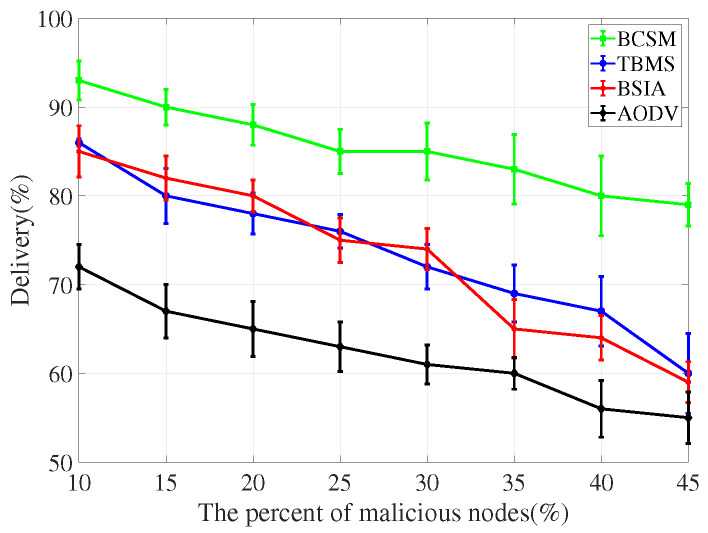
Delivery for high traffic density.

Figure 12 and Figure 13 plot the delivery rate in high and low traffic density scenarios, respectively. In either case, as the number of malicious nodes increases, the delivery rate decreases. AODV has a less packet delivery fraction because malicious nodes drop packets. Compared with other baseline methods, our proposed algorithm shows better performance in terms of delivery rate.

**Figure 14:**
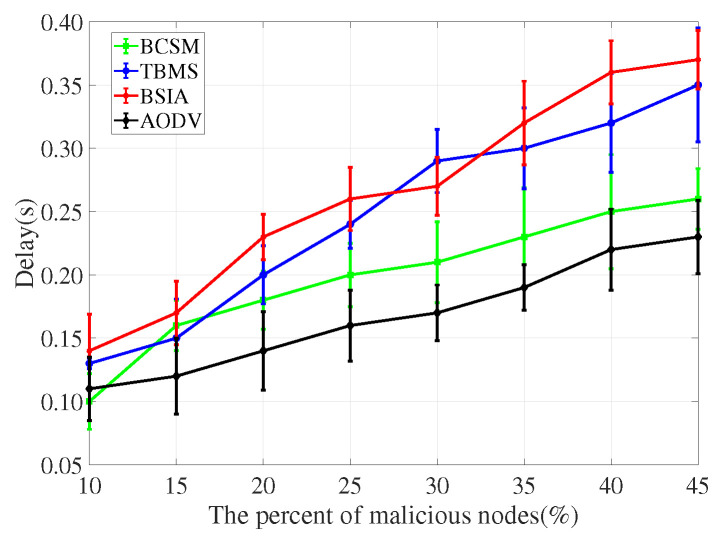
Delay for low traffic density.

**Figure 15:**
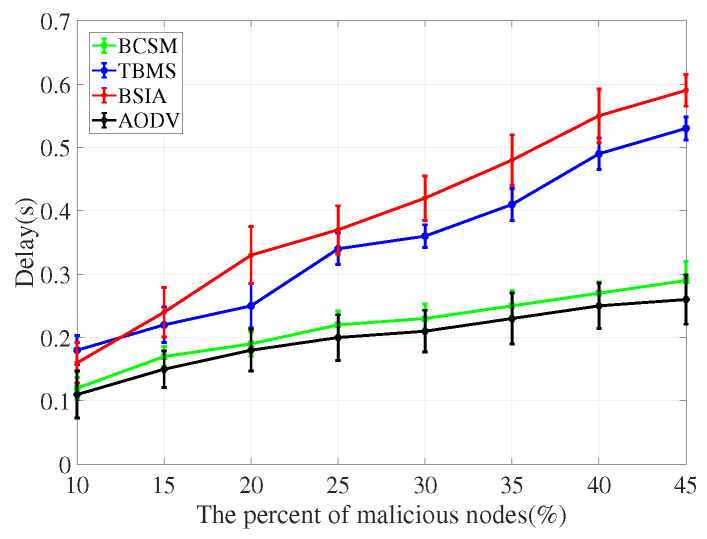
Delay for high traffic density.

Delay rate is the most important metric for assessing the network performance, as it depicts how an additional overhead of the security measure increases the delay in the process of routing. As shown in Figure 14 and Figure 15, AODV has the lowest delay rate because it does not consider any security measures. Compared with TBMS and BSIA, BCSM has a lower delay rate.

**Figure 16:**
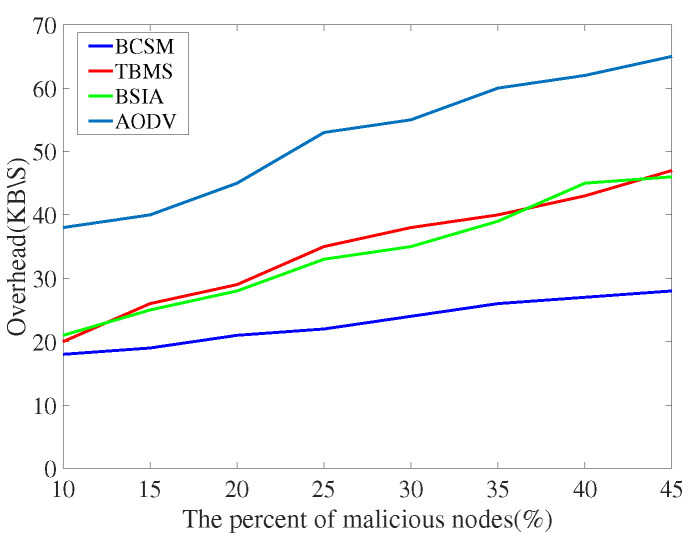
Overhead with low traffic density.

**Figure 17:**
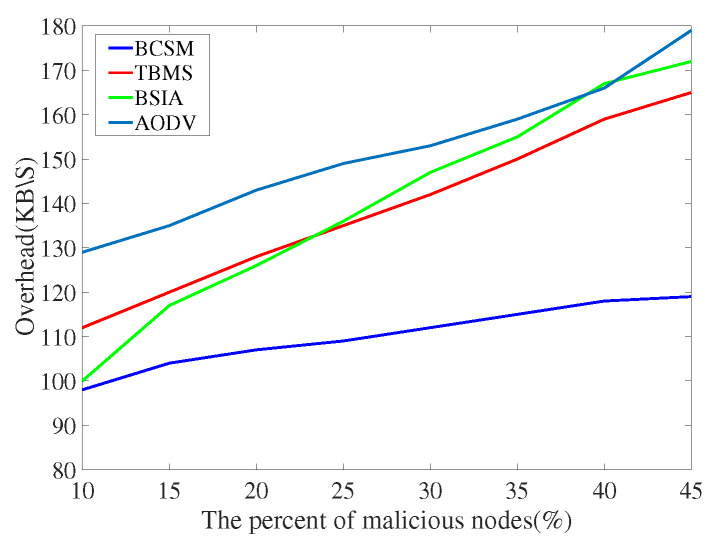
Overhead with high traffic density.

Figure 16 and Figure 17 show the comparison results of overhead rate. AODV has the lowest delay rate because it does not consider any security measures. In low traffic density, the overhead of our proposed algorithm is higher than that of other methods. In high traffic density, compared with other baseline methods, the overhead of our proposed algorithm is slightly higher than those of TBMS and BSIA. Obviously, BCSM is more suitable for urban traffic environments where high traffic density is almost the norm.

BCSM based on blockchain technology provides secure communications for CAs and RSUs. The experimental results show that BCSM effectively improves the efficacy of VANETs. Meanwhile, it achieves better performance in detecting forged messages and identifying malicious nodes.

## 6. Conclusions and Discussion

Detecting and identifying forged messages and malicious nodes in highly dynamic and complicated mobile networks is a challenging problem. In this paper, we proposed a security scheme based on blockchain technology for communication security in VANETs. The proposed scheme constructs two types of blockchains in VANETs: BCIR on RSUs and BCCA on CAs. An incentive consensus mechanism, which encourages RSUs to take active behaviors in VANETs, was designed for BCIR. BCCA is able to identify if a vehicle has a legitimate identity. The legitimacy of a message is evaluated, taking into account various factors, such as integrity of messages, reputation of the sender node, time effectiveness, and distance effectiveness. Meanwhile, the reputation of a node is decided by its communication behaviors. By analyzing a node’s communication behaviors, the scheme is able to identify whether or not the node is malicious. Moreover, the proposed scheme can protect the privacy of vehicles. Simulation-based experiments show that our scheme is feasible and effective for detecting malicious nodes and identifying forged messages in practical vehicle networks.

In the future, we plan to deepen our research to improve the accuracy of the scheme. We are currently in the process of building a traffic service platform to evaluate the performance of our scheme in real-life transportation networks. We will also consider additional security metrics for performance and robustness evaluation.

## Figures and Tables

**Table 1:** Message packet format.

Abbreviation	Values
SouID	ID of source node
RecID	ID of destination node
RecTime	Received Time
EventTime	The exact time of occured event
Event Type	I/II/III/IV
$Loc_{event}$	Location of event
$Loc_{vehicle}$	Location of source node
$TTL_{event}$	The life cycle of event
$Ran_{msg}$	Transmission range of the message

**Table 2:** Alert message classification.

Event ID	Event Type	${\mathrm{TTL}}_{\mathrm{event}}$	${\mathrm{Ran}}_{\mathrm{msg}}$
I	Road jam	$Th_{t-I}$	$Th_{d-I}$
II	Road accident	$Th_{t-II}$	$Th_{d-II}$
III	Icy road	$Th_{t-III}$	$Th_{d-III}$
IV	Road construction	$Th_{t-IV}$	$Th_{d-IV}$

**Table 3:** Simulation parameter settings.

Parameter Description	Value
Simulation area	4500 m × 3400 m
Simulation time	43,200 s
Mobility model	Shortest Path Map Based Movement
No. of groups	11
Number of nodes	100;400
Transmission rage	10 m
Node speed	2 m/s
Warm-up period	1000 s
Time to live	300
Buffer size	5M
RSU quantity	10
Routing scheme	ProphetRouter
$Egy_{o}$	0.5
$Th_{Egy}$	0.3
a	5
Pr	80%

## Data Availability

Not applicable.
